# Vest Chest Physiotherapy Airway Clearance is Associated with Nitric Oxide Metabolism

**DOI:** 10.1155/2013/291375

**Published:** 2013-11-21

**Authors:** Joseph H. Sisson, Todd A. Wyatt, Jacqueline A. Pavlik, Pawanjit S. Sarna, Peter J. Murphy

**Affiliations:** ^1^Pulmonary, Critical Care, Sleep & Allergy Division, Department of Internal Medicine, University of Nebraska Medical Center, Omaha, NE 68198-5910, USA; ^2^Research Service, Department of Veterans Affairs Omaha-Western Iowa Health Care System, 4101 Woolworth Avenue, Omaha, NE 68105, USA; ^3^Department of Environmental, Agricultural, and Occupational Health, College of Public Health, University of Nebraska Medical Center, Omaha, NE 68198-7850, USA

## Abstract

*Background*. Vest chest physiotherapy (VCPT) enhances airway clearance in cystic fibrosis (CF) by an unknown mechanism. Because cilia are sensitive to nitric oxide (NO), we hypothesized that VCPT enhances clearance by changing NO metabolism. *Methods*. Both normal subjects and stable CF subjects had pre- and post-VCPT airway clearance assessed using nasal saccharin transit time (NSTT) followed by a collection of exhaled breath condensate (EBC) analyzed for NO metabolites (NO_*x*_). *Results*. VCPT shorted NSTT by 35% in normal and stable CF subjects with no difference observed between the groups. EBC NO_*x*_ concentrations decreased 68% in control subjects after VCPT (before = 115 ± 32 **μ**M versus after = 37 ± 17 **μ**M; *P* < 0.002). CF subjects had a trend toward lower EBC NO_*x*_. *Conclusion*. We found an association between VCPT-stimulated clearance and exhaled NO_*x*_ levels in human subjects. We speculate that VCPT stimulates clearance via increased NO metabolism.

## 1. Introduction

Percussive chest physiotherapy (CPT) is the principal treatment that patients use to facilitate clearance of airway secretions with cystic fibrosis (CF) or other causes of bronchiectasis. Patients have historically used various forms of clapping or mechanical percussion to accomplish this. A number of devices, collectively referred to as vest chest physiotherapy (VCPT), are now available that allow patients to perform airway clearance without the aid of a second person to apply the therapy [1]. Several studies demonstrate the superiority of chest physiotherapy over no chest physiotherapy with regard to clinical outcomes [2]. Chest physiotherapy increases mucus clearance as assessed by mucus volume measurements [3].

The mechanism by which percussive chest physiotherapy modalities enhance airway clearance is not known. Clinicians hypothesize that percussion and shaking loosen adherent mucus and biofilms from the airway surface, making it easier for cough clearance to remove them from the airways. Alternatively, a number of investigators have shown that mechanical stimulation of certain tissue types results in increased epithelial cell release of NO [4].

Because mechanical stimulation increases NO release and airway clearance [5], we hypothesized that VCPT alters metabolism of NO, as measured by oxides of nitrogen (NO_*x*_) release from the human airway, and is associated with enhanced airway clearance. To test these hypotheses, we measured nasal saccharin transit time (NSTT) and NO_*x*_ in exhaled breath condensate (EBC) in subjects with and without CF, before and after a therapy session with the VCPT.

## 2. Materials and Methods

This study was conducted at the University of Nebraska Medical Center (UNMC), Omaha, Nebraska, and approved by the UNMC Institutional Review Board. Informed consent was obtained from each subject.

### 2.1. Experimental Design

**Figure 1:**
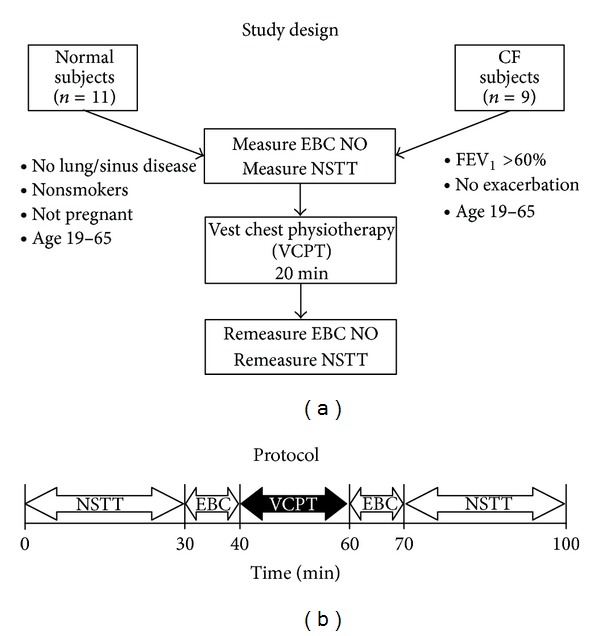
Diagram of the study design.

The protocol for the study is outlined in Figure 1. Control patients were normal, healthy volunteer adults aged 19–65 years in good health (Table 1). Patients qualified for inclusion if they had a documented diagnosis of cystic fibrosis, FEV1 greater than 60%, and were adults aged 19–65 years. The majority of the CF patient group had been exposed to vest physiotherapy in the past, but none had undergone a treatment within 24 hours of participation in the study. All patients and control subjects abstained from drinking alcohol for at least 24 hours prior to the study. Control subjects were excluded if they had a history of lung or sinus disease, had ever smoked, or were pregnant. CF subjects were excluded if they were smokers, were pregnant, or had evidence of acute pulmonary exacerbation of their disease. For the purposes of our study, we defined a CF pulmonary exacerbation as an increase in cough, sputum, or new dyspnea or other symptoms sufficient to justify acute treatment with an antibiotic delivered by any route.

### 2.2. Subject Selection and Enrollment

We solicited patients for participation in the study during routine visits for periodic CF care. Patients were allowed to participate only if they and their treating physician agreed that they were near their baseline state of health with no evidence of CF pulmonary exacerbation or other acute illness [6]. We conducted the study over a period of four months.

After informed consent, subjects underwent nasal saccharin transit time (NSTT) testing (see below), followed by a collection of the pre-percussion exhaled breath condensate (EBC) for 10 minutes during tidal breathing. Each subject underwent a VCPT per protocol outlined below. The post-NSTT procedure immediately followed the collection of postpercussion EBC for 10 minutes, both in a manner identical to the prepercussion collection and testing (but in reverse order; Figure 1).

### 2.3. Nasal Saccharin Transit Time

Under direct visualization, a particle (5 mg) of saccharin measuring approximately 0.5 mm in diameter was placed onto one of the inferior nasal turbinates of each participant as described [7]. The elapsed time was recorded from the placement of the saccharin particle to the first taste of sweetness. Measurement of NSTT was performed both before and after VCPT.

### 2.4. Exhaled Breath Condensate

Samples of exhaled breath condensate were collected by tidal breathing for 10 minutes into an EcoScreen, (Jaeger, Würzburg, Germany) cooled system forming a condensate. To prevent contamination from nasal breathing, a nose clip was worn throughout collection of the EBC. Samples were divided into 500 *μ*L aliquots and stored at −70°C for nitric oxide analysis at a later date [8].

### 2.5. Vest Chest Physiotherapy

Subjects underwent a 20-minute VCPT session with the Model 104 Air-Pulse Generator (Hill-Rom Corporate, St. Paul, MN). The vest was rapidly inflated and deflated to the maximum-tolerated pressureat a frequency of 10–15 Hz for 20 minutes.

### 2.6. Measurement of NO_*x*_

Oxides of nitrogen were measured from the frozen EBC samples using a Sievers NOA 280 chemiluminescence analyzer (GE Analytical Instruments, Boulder, CO). The analyzer uses a vanadium chloride reduction to convert NO_*x*_ back to nitric oxide. In this system, both nitrites and nitrates were assayed, as conversion products of exhaled breath NO. Calculation of NO_*x*_ concentration was extrapolated from a standard curve of 1 nM to 100 **μ**M sodium nitrate. For each sample, triplicate injections were run on two different days for each data point. Significance was determined by paired *t*-test with *P* value <0.05.

## 3. Results

### 3.1. Nasal Saccharin Transit Time (NSTT)

**Figure 2:**
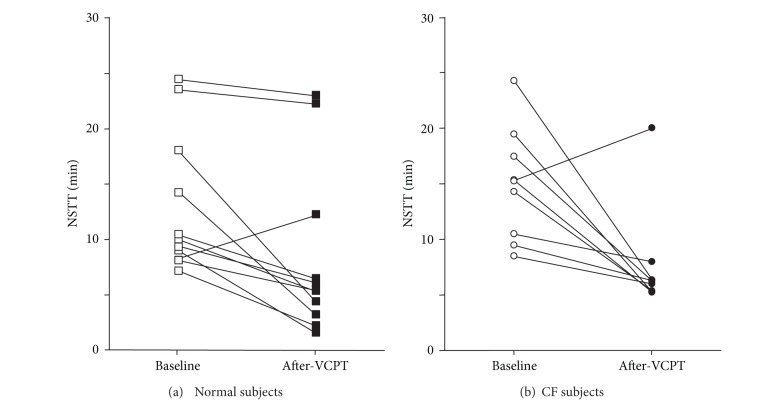
*Nasal saccharin transit time (NSTT) after vest chest physiotherapy (VCPT). *(a) All but one normal subject demonstrated a decrease in NSTT following vest therapy. (b) All CF subjects except one sustained a decrease in NSTT after VCPT when compared to pretreatment values.

**Figure 3:**
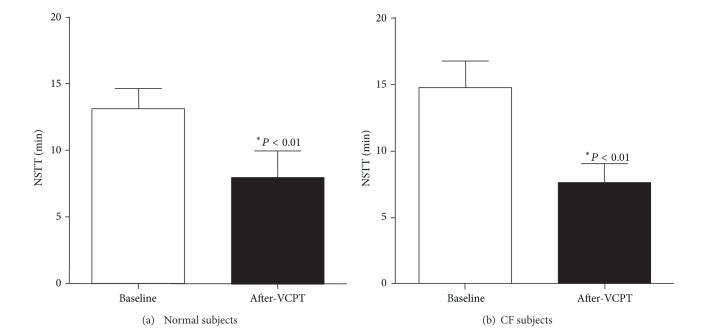
*Aggregate nasal saccharin transit time after VCPT. *(a) Normal subjects demonstrated a 35% decrease in NSTT following VCPT as compared to pretreatment NSTT (13.0 ± 1.9 minutes versus 8.4 ± 2.3 min; *P* < 0.01). (b) In CF subjects, we observed a similar 38% decrease in NSTT after VCPT when compared to pretreatment values (baseline = 15.5 ± 1.6 min versus after vest = 8.3 ± 1.6 min; *P* < 0.01).

NSTT was significantly reduced after VCPT in normal subjects (Figure 2(a)) meaning that clearance was enhanced. All but one normal subject demonstrated a decrease in NSTT following vest therapy (Figure 2(a)). In aggregate, normal subjects demonstrated a 35% decrease in NSTT following VCPT as compared to pretreatment NSTT (Figure 3(a); 13.0 ± 1.9 minutes versus 8.4 ± 2.3 min; *P* < 0.01). We compared these results with nasal clearance in patients with cystic fibrosis. We observed a similar 38% decrease in NSTT after VCPT when compared to pretreatment values (Figures 2(b) and 3(b); baseline = 15.5 ± 1.6 min versus post-VCPT = 8.3 ± 1.6 min; *P* < 0.01). As with the normal subjects, all CF subjects, save one, sustained a decrease in NSTT after VCPT when compared to pretreatment values (Figure 2(b)). We observed no significant difference in either baseline or post-vest CPT NSTT values between normal subjects and CF patients (Figure 3). 

### 3.2. Exhaled Breath Condensate (EBC) Oxides of Nitrogen (NO_*x*_)

**Figure 4:**
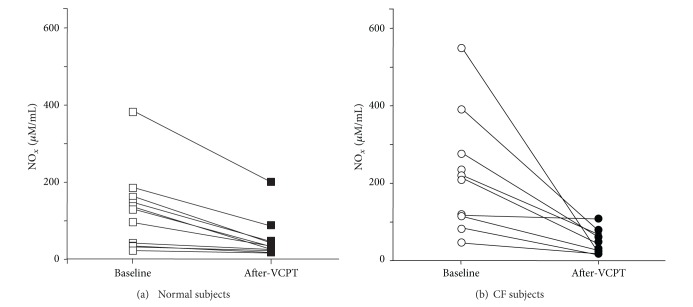
*Exhaled breath condensate (EBC) oxides of nitrogen ( N* *O*_*x*_ *) following VCBT. *(a) All 11 normal subjects sustained a decrease in EBC NO_*x*_ concentrations. (b) All nine cystic fibrosis subjects also sustained a decrease in EBC NO_*x*_ concentrations.

**Figure 5:**
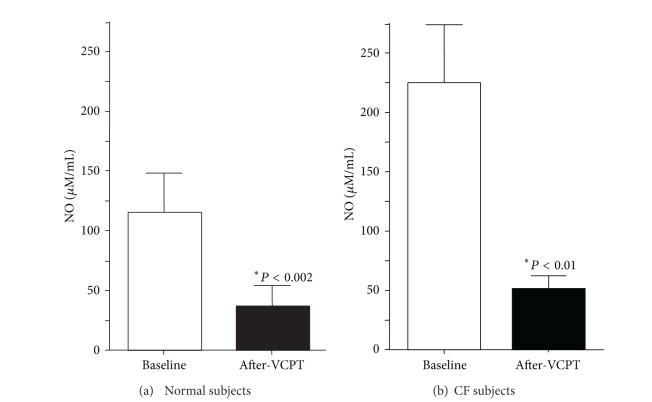
*Aggregate exhaled breath condensate oxides of nitrogen following VCBT. *(a) EBC NO_*x*_ concentrations decreased 68% in control subjects after VCPT compared to baseline, pretreatment values (baseline = 115 ± 32 **μ*M* versus post-VCPT = 37 ± 17 **μ*M*; *P* < 0.002). (b) CF subjects showed a significant decrease in EBC NO_*x*_ after vest percussion treatment (baseline = 224 ± 55 **μ*M* versus post-vest = 52 ± 11 **μ*M*; *P* < 0.01).

Because cilia stimulation is associated with nitric oxide (NO) production, we measured NO_*x*_ concentration in exhaled breath condensate (EBC) before and after VCPT. We found that EBC NO_*x*_ concentrations decreased 68% in control subjects after VCPT compared to baseline, pretreatment values (Figure 5(a); baseline = 115 ± 32 **μ**M versus post-vest = 37 ± 17 **μ**M; *P* < 0.002). Although with different degrees, all 11 normal subjects sustained a decrease in EBC NO_*x*_ concentrations (Figure 4(a)). We compared these results in normal subjects to patients with stable cystic fibrosis and found a similar result (Figure 4(b)). All patients with CF showed a significant decrease in EBC NO_*x*_ after VCPT (Figure 5(b); baseline = 224 ± 55 **μ**M versus post-vest = 52 ± 11 **μ**M; *P* < 0.01). As with NSTT, we saw no difference in baseline or post-VCPT EBC NO_*x*_ values between normal subjects and subjects with stable CF (*P* = 0.09). There was not a statistically significant correlation between the decrease in NSTT and the decrease in EBC [NO_*x*_].

## 4. Discussion

We hypothesized that mechanical stimulation with VCPT alters NO_*x*_ metabolism, which stimulates mucociliary clearance. To test this hypothesis, we measured nasal saccharin transit time and exhaled breath condensate NO_*x*_ concentrations in healthy normal adult subjects and adults with stable cystic fibrosis before and after VCPT. In this model, we use NSTT as a surrogate of mucociliary clearance.

To our knowledge, this is the first study to associate changes in airway mucociliary clearance with changes in exhaled NO_*x*_ in human subjects. Our results indicate that VCPT applied to both normal and stable CF subjects significantly increased nasal mucociliary clearance. A single VCPT treatment increased nasal clearance in 18 out of 20 (90%) subjects tested. In aggregate, we found that a vest treatment increased nasal clearance by approximately 35% in both normal and stable CF subjects. Although our study groups were small in number, the effect of percussion on nasal clearance was quite striking with both biologically relevant and statistically significant changes in NSTT.

We originally designed our study to address the mechanism of enhanced clearance by VCPT. We chose to explore the role of NO that might have in enhanced clearance based on *in vitro *studies of cilia that date back to 1993 demonstrating that increases of CBF by multiple stimuli require NO production [9]. Many investigators have corroborated these findings in several different systems [10–12]. In this context, we originally hypothesized that VCPT-enhanced clearance would increase exhaled NO_*x*_ concentrations. Our results are counterintuitive and exactly the opposite of our expectations regarding the effect of mechanical stimulation via VCPT on exhaled NO_*x*_. In every case, VCPT is associated with dramatic reduction in NO_*x*_ concentrations. We speculate that mechanical shaking with the VCPT device enhances mucus clearance by stimulating calcium release from airway epithelial cells, resulting in calmodulin activation of eNOS with resultant production of NO in the airway epithelial cell. If NO production increases with mechanical stimulation, then we have to reconcile our observation that EBC NO_*x*_ decreased following VCPT. An alternative explanation for the difference between our findings in this study and those published with NO_*x*_ measurements performed in ciliated cells *in vitro* is that our study focused on the metabolites of NO that were exhaled into the EBC while the *in vitro* studies measured the concentration of NO_*x*_ present in the media. It is likely that “off-gassing” of NO is the basis of NO_*x*_ in EBC [13], whereas, *in vitro* studies focus on dissolved and non-volatile metabolites of NO present in the media.

The decrease in exhaled NO_*x*_ may be due to increased utilization of NO within the ciliated epithelial cell. In this scenario, the enhanced binding of NO to guanylyl cyclase would provide the signal for elevated cGMP, activation of PKG, and stimulated cilia beating. It is also possible that the increased mucus flow from more distal airways creates a greater barrier to NO diffusion from the airway epithelium during percussion therapy than that present in the resting state. Alternatively, mechanical stimulation through percussion may alter the metabolism of NO through an unknown mechanism.

The literature has clearly established that mechanical stress alone can stimulate cilia beating in both *in vitro * [14] and* in vivo* [15] models. The mechanism of such mechanical stimulation may involve an increase in cytoplasmic calcium resulting from either extracellular calcium influx or inositol-1′,4′,5-triphosphate-mediated internal calcium release [16, 17]. The calcium pathway is likely independent of the NO-cGMP-PKG stimulation of cilia beating in an ovine model [18], but others have shown a cGMP-dependent component to calcium-stimulated cilia beat in rabbit airway [18]. Using human nasal ciliated cells, Alberty et al. demonstrated that mechanical stimulation of cilia could take place in the presence of an NO inhibitor, suggesting that the mechanical induced increases in CBF were independent of endogenous NO production [19]. Extrapolating from these observations, mechanical stimulation of mucociliary transport in response to VCPT in our study could be independent of NO. However, the significant *decrease* in EBC NO_*x*_ levels in response to mechanical stimulation in both study groups suggests a distinct mechanical impact on NO metabolism and release from the lung.

An obvious question that arises from our findings is as follows: *how does chest percussion alter nasal clearance*? The vest percussion apparatus is applied to the chest with the intent of oscillating the lower airways. We observed, however, that the head and neck clearly oscillated in sync with the chest during VCPT. Furthermore, increased nasal discharge typically occurs during percussion treatment consistent with increased nasal clearance (unpublished results). From these observations we surmise that the nasal cavity, in addition to the lower airways, is vigorously shaken during VCPT and results in stimulated nasal mucociliary clearance.

Another important question is as follows: *does nasal clearance correlate with lung airway clearance?* Because VCPT is typically directed at patients with lung airway clearance disorders such as bronchiectasis, we can only cautiously extrapolate our findings with nasal clearance to lung clearance. We did not directly measure lung clearance due to the cost and exposure to radionuclides involved in such studies. The NSTT is, however, a direct functional measure of mucociliary clearance of the upper airway. Because the vest is thought to enhance lung airway clearance, it is highly likely that both nasal and lung clearance are stimulated by VCPT. 

We also note that EBC NO_*x*_ tended to be *higher* in CF subjects than in control subjects (Figure 5), which differs from previous studies [20]. While we acknowledge that the NO_*x*_ difference between normal and CF subjects was not statistically different, the best explanation for this disparity is that the earlier study measured only direct exhaled NO, which does not include nitric oxide breakdown products. In contrast, exhaled breath condensate contains the stable conversion compounds of NO, nitrites, and nitrates. Our findings measuring NO_*x*_ concentrations in EBC contrast with other studies that measured only the exhaled free radical NO.

## 5. Conclusions

In summary, we found that VCPT meaningfully enhanced airway clearance, as measured by NSTT in all subjects. In addition, we found that exhaled breath NO_*x*_ levels significantly *decreased* following VCPT. These findings suggest an association between VCPT clearance changes and altered airway NO_*x*_ levels consistent with a role for NO metabolism in the regulation of mucociliary clearance. These findings suggest the need to further study the mechanisms of increased airway clearance during chest physiotherapy to establish the role nitric oxide plays in regulating this response.

## Figures and Tables

**Table 1:** Study subject demographics.

	Normal subjects (*n* = 11)	Cystic fibrosis subjects (*n* = 9)
Male	6	4
Female	5	5
Age	30–51	21–44
